# Complete genome sequence of the gliding, heparinolytic *Pedobacter saltans* type strain (113^T^)

**DOI:** 10.4056/sigs.2154937

**Published:** 2011-09-23

**Authors:** Konstantinos Liolios, Johannes Sikorski, Meagan Lu, Matt Nolan, Alla Lapidus, Susan Lucas, Nancy Hammon, Shweta Deshpande, Jan-Fang Cheng, Roxanne Tapia, Cliff Han, Lynne Goodwin, Sam Pitluck, Marcel Huntemann, Natalia Ivanova, Ioanna Pagani, Konstantinos Mavromatis, Galina Ovchinikova, Amrita Pati, Amy Chen, Krishna Palaniappan, Miriam Land, Loren Hauser, Evelyne-Marie Brambilla, Oleg Kotsyurbenko, Manfred Rohde, Brian J. Tindall, Birte Abt, Markus Göker, John C. Detter, Tanja Woyke, James Bristow, Jonathan A. Eisen, Victor Markowitz, Philip Hugenholtz, Hans-Peter Klenk, Nikos C. Kyrpides

**Affiliations:** 1 DOE Joint Genome Institute, Walnut Creek, California, USA; 2 DSMZ - German Collection of Microorganisms and Cell Cultures GmbH, Braunschweig, Germany; 3 Los Alamos National Laboratory, Bioscience Division, Los Alamos, New Mexico, USA; 4 Biological Data Management and Technology Center, Lawrence Berkeley National Laboratory, Berkeley, California, USA; 5 Oak Ridge National Laboratory, Oak Ridge, Tennessee, USA; 6 Technical University of Braunschweig, Institute for Microbiology, Braunschweig, Germany; 7 Lomonosov Moscow State University, Biological Department, Moscow, Russia; 8 HZI – Helmholtz Centre for Infection Research, Braunschweig, Germany; 9 University of California Davis Genome Center, Davis, California, USA; 10 Australian Centre for Ecogenomics, School of Chemistry and Molecular Biosciences, The University of Queensland, Brisbane, Australia

**Keywords:** strictly aerobic, gliding motility, Gram-negative, heparinolytic, mesophilic, chemoorganotrophic, *Sphingobacteriaceae*, GEBA

## Abstract

*Pedobacter saltans* Steyn *et al.* 1998 is one of currently 32 species in the genus *Pedobacter* within the family *Sphingobacteriaceae*. The species is of interest for its isolated location in the tree of life. Like other members of the genus *P. saltans* is heparinolytic. Cells of *P. saltans* show a peculiar gliding, dancing motility and can be distinguished from other *Pedobacter* strains by their ability to utilize glycerol and the inability to assimilate D-cellobiose. The genome presented here is only the second completed genome sequence of a type strain from a member of the family *Sphingobacteriaceae* to be published. The 4,635,236 bp long genome with its 3,854 protein-coding and 67 RNA genes consists of one chromosome, and is a part of the *** G****enomic* *** E****ncyclopedia of* *** B****acteria and* *** A****rchaea * project.

## Introduction

Strain 113^T^ (= DSM 12145 = LMG 10337 = NBRC 100064) is the type strain of the species *Pedobacter saltans* [1], one of currently 32 validly named species in the genus *Pedobacter* [2]. We prefer to use here the strain designation ‘113’ as originally published by Steyn *et al*. in 1992 [3] and as also shown in the LMG online catalogue [4] and in the StrainInfo database [5] over the designation ‘LMG 10337^T^’ which was later used for the description of the species by the same authors [1]. The genus name is derived from the Latinized Greek word 'pedon' meaning 'the ground, earth' and the Neo-Latin word 'bacter' meaning 'rod', yielding '*Pedobacter*', the 'rod from soil' [1]. The species epithet is derived from the Latin word 'saltare' meaning 'to dance', yielding 'saltans', referring to the gliding motility of the strain' [1]. *P. saltans* strain 113^T^ was isolated from soil in Iceland; several more strains belonging to the species were isolated from soil in Iceland, Belgium (Brussels) and Germany (Rüdesheim) [1]. Members of the genus *Pedobacter* were isolated from various environments including different soils [1,6-10], water [11-13], a nitrifying inoculum [14], glaciers [15,16], fish [1] and compost [17]. Here we present a summary classification and a set of features for *P. saltans* strain 113^T^, together with the description of the complete genome sequence and the genome annotation.

## Classification and features

A representative genomic 16S rRNA sequence of strain 113^T^ was compared using NCBI BLAST [18,19] under default settings (e.g., considering only the high-scoring segment pairs (HSPs) from the best 250 hits) with the most recent release of the Greengenes database [20] and the relative frequencies of taxa and keywords (reduced to their stem [21]) were determined, weighted by BLAST scores. The most frequently occurring genera were *Pedobacter* (53.4%), *Sphingobacterium* (33.3%), *Mucilaginibacter* (5.0%), *Flavobacterium* (4.1%) and *'Sphingoterrabacterium'* (2.1%) (95 hits in total). Regarding the two hits to sequences from members of the species, the average identity within HSPs was 99.7%, whereas the average coverage by HSPs was 97.6%. Regarding the 20 hits to sequences from other members of the genus, the average identity within HSPs was 92.8%, whereas the average coverage by HSPs was 84.1%. Among all other species, the one yielding the highest score was *Pedobacter lentus* (EF446146), which corresponded to an identity of 93.2% and an HSP coverage of 93.4%. (Note that the Greengenes database uses the INSDC (= EMBL/NCBI/DDBJ) annotation, which is not an authoritative source for nomenclature or classification.) The highest-scoring environmental sequence was HM008274 ('anodic biomass air-cathode single chamber microbial fuel cell clone 9week.anode.2'), which showed an identity of 94.6% and an HSP coverage of 83.8%. The most frequently occurring keywords within the labels of environmental samples which yielded hits were 'skin' (8.6%), 'fossa' (4.2%), 'poplit' (2.2%), 'soil' (2.2%) and 'forearm, volar' (2.0%) (152 hits in total). Interestingly, several of the most frequent keywords relate to a mammalian or clinical habitats, which may allude to some yet unknown ecological features of *P. saltans*, taking into account that all known isolates are from soil in different countries [1]. However, environmental samples which yielded hits of a higher score than the highest scoring species were not found.

**Figure 1:**
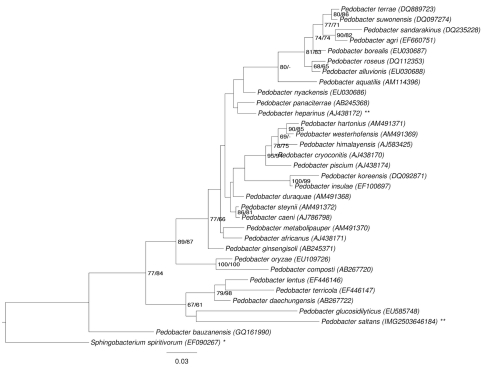
Phylogenetic tree highlighting the position of *P. saltans* relative to the other type strains within the genus *Pedobacter*. The tree was inferred from 1,402 aligned characters [22,23] of the 16S rRNA gene sequence under the maximum-likelihood (ML) criterion [24] and rooted with the type strain of the type species of the family *Sphingobacteriaceae* [25]. The branches are scaled in terms of the expected number of substitutions per site. Numbers adjacent to the branches are support values from 550 ML bootstrap replicates [26] (left) and from 1,000 maximum-parsimony bootstrap replicates [27] (right) if larger than 60%. Lineages with type strain genome sequencing projects registered in GOLD [28] as unpublished are marked with one asterisk, those listed as published with two asterisks [29]. Note that the taxon selection used in this figure does not allow conclusions about the monophyly of the genus *Pedobacter*. In an expanded analysis also including the genera *Mucilaginibacter* and *Nubsella* (data not shown), neither the Kishino-Hasegawa test as implemented in PAUP* [27] in conjunction with the maximum-parsimony criterion nor the Shimodaira-Hasegawa test as implemented in RAxML [24] in conjunction with the ML criterion indicated a significant difference between the respective globally best tree and the best tree after constraining for the monophyly of all four genera. (See, e.g. chapter 21 in [30] for an in-depth description of such paired-site tests.)

Figure 1 shows the phylogenetic neighborhood of *P. saltans* in a 16S rRNA based tree. The sequences of the four 16S rRNA gene copies in the genome differ from each other by up to one nucleotide, and differ by up to three nucleotides from the previously published 16S rRNA sequence (AJ438173).

**Figure 2:**
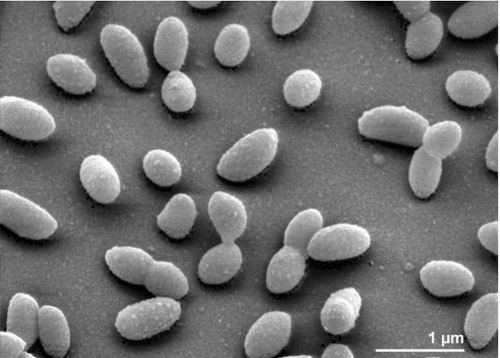
Scanning electron micrograph of *P. saltans* strain 113^T^

The cells of *P. saltans* are short rods (0.5 × 0.7-1.0 µm) with rounded or slightly tapering ends (Figure 2) [1]. Three of the four strains belonging to *P. saltans* were described as being motile *via* gliding [1]. *P. saltans* cells strain Gram-negative and are non-spore-forming (Table 1). Strain 113^T^ is strictly aerobic and chemoorganotrophic [1]. Colonies on modified TSA are smooth, light yellow to yellow, translucent, round, 2-5 mm in diameter, convex to slightly umbonate with entire margins [1]. On nutrient agar colonies are smooth, yellow, round, 2-4 mm in diameter, convex with entire to scalloped margins [1]. The temperature range for growth is normally between 5°C and 30°C [1]. The biochemical features and antibiotic resistance of *P. saltans* has been described previously [1]. Strain 113^T^ produces acetoin from sodium pyruvate, degrades chondroitin sulfate and hydrolyzes aesculin. It grows on heparin, which is degraded by inducible enzymes. Good growth occurs on nutrient agar or on regular or modified TSA. *P. saltans* does not produce H_2_S from thiosulfate and does not grow on MacConkey agar [1]. *P. saltans* can be differentiated phenotypically from other *Pedobacter* species by its inability to assimilate D-cellobiose and the ability to utilize glycerol. The organism does not reduce nitrate [1].

**Table 1:** Classification and general features of *P. saltans* strain 113^T^ according to the MIGS recommendations [31] and the NamesforLife database [2]

MIGS ID	Property	Term	Evidence code
	Current classification	Domain *Bacteria*	TAS [32]
		Phylum “*Bacteroidetes*”	TAS [33]
		Class “*Sphingobacteria*”	TAS [34]
		Order “*Sphingobacteriales*”	TAS [34]
		Family *Sphingobacteriaceae*	TAS [1]
		Genus *Pedobacter*	TAS [1]
		Species *Pedobacter saltans*	TAS [1,11,12,14]
		Type strain 113	TAS [1,3]
	Gram stain	negative	TAS [1]
	Cell shape	short rods with rounded, slightly tapering ends	TAS [1]
	Motility	gliding	TAS [1]
	Sporulation	none	TAS [1]
	Temperature range	5°C–30°C	TAS [1]
	Optimum temperature	not reported	
	Salinity	not reported	
MIGS-22	Oxygen requirement	strictly aerobic	TAS [1]
	Carbon source	carbohydrates, some alcohols and glycosides	TAS [1]
	Energy metabolism	chemoorganotroph	TAS [1]
MIGS-6	Habitat	soil	TAS [1]
MIGS-15	Biotic relationship	free-living	NAS
MIGS-14	Pathogenicity	none	NAS
	Biosafety level	1	TAS [35]
	Isolation	soil	TAS [3]
MIGS-4	Geographic location	Iceland	TAS [1,3]
MIGS-5	Sample collection time	1992 or before	TAS [1,3]
MIGS-4.1	Latitude	not reported	
MIGS-4.2	Longitude	not reported	
MIGS-4.3	Depth	not reported	
MIGS-4.4	Altitude	not reported	

Evidence codes - TAS: Traceable Author Statement (i.e., a direct report exists in the literature); NAS: Non-traceable Author Statement (i.e., not directly observed for the living, isolated sample, but based on a generally accepted property for the species, or anecdotal evidence). These evidence codes are from of the Gene Ontology project [36].

### Chemotaxonomy

The cell wall of the members of the genus *Pedobacter* contain sphingolipids and menaquinone-7 as the predominant menaquinone system [11-13]. Strain 113^T^ contains the following fatty acids: *iso*-C_15:0_ (31.4%), C_16:1ω7c_ (19.6%), *iso*-C_17:0 3-OH_ (12.7%), *iso*-C_15:0 2-OH_ (8.9%), *iso*-C_17:1ω9c_ (6.6%), C_16:0_ (4.0%), *anteiso*-C_15:0_ (2.9%), *iso*-C_15:0 3-OH_ (2.8%), C_15:0_ (1.4%), C_15:1ω6c_ (1.4%), and C_16:1ω7c_ (19.6%) which are acids typical of the genus. It also contains traces of C_14:0_, C_16:1ω5c_,  and C_16:0 3-OH_ [1].

## Genome sequencing and annotation

### Genome project history

This organism was selected for sequencing on the basis of its phylogenetic position [37], and is part of the *** G****enomic* *** E****ncyclopedia of* *** B****acteria and* *** A****rchaea * project [38]. The genome project is deposited in the Genome OnLine Database [28] and the complete genome sequence is deposited in GenBank. Sequencing, finishing and annotation were performed by the DOE Joint Genome Institute (JGI). A summary of the project information is shown in Table 2.

**Table 2:** Genome sequencing project information

**MIGS ID**	**Property**	**Term**
MIGS-31	Finishing quality	Finished
MIGS-28	Libraries used	Tree genomic libraries: one 454 pyrosequence standard library, one 454 PE library (7.7 kb insert size), one Illumina library
MIGS-29	Sequencing platforms	Illumina GAii, 454 GS FLX Titanium
MIGS-31.2	Sequencing coverage	645.0 × Illumina; 19.5 × pyrosequence
MIGS-30	Assemblers	Newbler version 2.3, Velvet version 0.7.63, phrap version SPS - 4.24
MIGS-32	Gene calling method	Prodigal 1.4, GenePRIMP
	INSDC ID	CP002545
	Genbank Date of Release	March 2, 2011
	GOLD ID	Gc01673
	NCBI project ID	49337
	Database: IMG-GEBA	649633082
MIGS-13	Source material identifier	DSM 12145
	Project relevance	Tree of Life, GEBA

### Growth conditions and DNA isolation

*P. saltans* 113^T^ (DSM 12145), was grown in DSMZ medium 605 (Nutrient agar (Oxoid CM3)) [39] at 28°C. DNA was isolated from 0.5-1 g of cell paste using Jetflex Genomic DNA Purification Kit (GENOMED 600100), modified by 1 hour incubation at 58°C with 20 µl proteinase for improved cell lysis. DNA is available through the DNA Bank Network [40].

### Genome sequencing and assembly

The genome was sequenced using a combination of Illumina and 454 sequencing platforms. All general aspects of library construction and sequencing can be found at the JGI website [41]. Pyrosequencing reads were assembled using the Newbler assembler (Roche). The initial Newbler assembly consisting of 44 contigs in two scaffolds was converted into a phrap [42] assembly by making fake reads from the consensus, to collect the read pairs in the 454 paired end library. Illumina sequencing data (6,233.8 Mb) was assembled with Velvet [43] and the consensus sequences were shredded into 1.5 kb overlapped fake reads and assembled together with the 454 data. The 454 draft assembly was based on 112.7 Mb 454 draft data and all of the 454 paired end data. Newbler parameters are -consed -a 50 -l 350 -g -m -ml 20. The Phred/Phrap/Consed software package [42] was used for sequence assembly and quality assessment in the subsequent finishing process. After the shotgun stage, reads were assembled with parallel phrap (High Performance Software, LLC). Possible mis-assemblies were corrected with gapResolution [41], Dupfinisher [44], or sequencing cloned bridging PCR fragments with subcloning. Gaps between contigs were closed by editing in Consed, by PCR and by Bubble PCR primer walks (J.-F. Chang, unpublished). A total of 205 additional reactions were necessary to close gaps and to raise the quality of the finished sequence. Illumina reads were also used to correct potential base errors and increase consensus quality using a software Polisher developed at JGI [45]. The error rate of the completed genome sequence is less than 1 in 100,000. Together, the combination of the Illumina and 454 sequencing platforms provided 664.5 × coverage of the genome. The final assembly contained 205,963 pyrosequence and 82,382,711 Illumina reads.

### Genome annotation

Genes were identified using Prodigal [46] as part of the Oak Ridge National Laboratory genome annotation pipeline, followed by a round of manual curation using the JGI GenePRIMP pipeline [47]. The predicted CDSs were translated and used to search the National Center for Biotechnology Information (NCBI) non-redundant database, UniProt, TIGR-Fam, Pfam, PRIAM, KEGG, COG, and InterPro databases. Additional gene prediction analysis and functional annotation was performed within the Integrated Microbial Genomes - Expert Review (IMG-ER) platform [48].

## Genome properties

**Figure 3:**
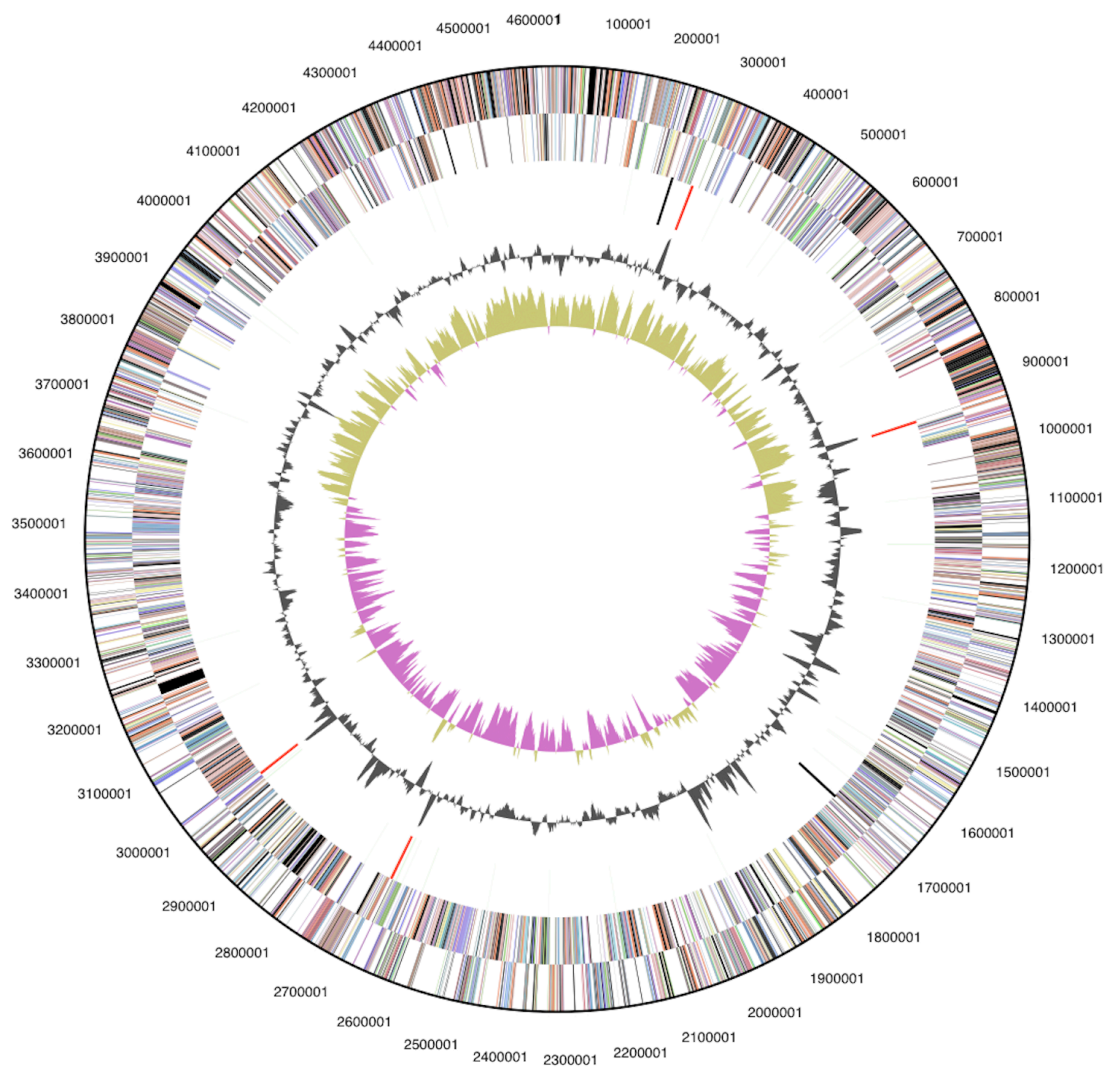
Graphical circular map of genome. From outside to the center: Genes on forward strand (color by COG categories), Genes on reverse strand (color by COG categories), RNA genes (tRNAs green, rRNAs red, other RNAs black), GC content, GC skew.

The genome consists of a 4,635,236 bp long chromosome with a G + C content of 36.6% (Table 3 and Figure 3). Of the 3,921 genes predicted, 3,854 were protein-coding genes, and 67 RNAs; 62 pseudogenes were also identified. The majority of the protein-coding genes (64.8%) were assigned a putative function while the remaining ones were annotated as hypothetical proteins. The distribution of genes into COGs functional categories is presented in Table 4.

**Table 3:** Genome Statistics

**Attribute**	**Value**	**% of Total**
Genome size (bp)	4,635,236	100.00%
DNA coding region (bp)	4,149,395	89.52%
DNA G+C content (bp)	1,695,689	36.58%
Number of replicons	1	
Extrachromosomal elements	0	
Total genes	3,921	100.00%
RNA genes	67	1.71%
rRNA operons	4	
Protein-coding genes	3,854	98.29%
Pseudo genes	62	1.58%
Genes with function prediction	2,539	64.75%
Genes in paralog clusters	87	2.22%
Genes assigned to COGs	2,644	67.43%
Genes assigned Pfam domains	2,757	70.31%
Genes with signal peptides	1,646	41.98%
Genes with transmembrane helices	898	22.90%
CRISPR repeats	0	

**Table 4:** Number of genes associated with the general COG functional categories

**Code**	**value**	**%age**	**Description**
J	158	5.5	Translation, ribosomal structure and biogenesis
A	0	0.0	RNA processing and modification
K	175	6.1	Transcription
L	142	5.0	Replication, recombination and repair
B	1	0.0	Chromatin structure and dynamics
D	26	0.9	Cell cycle control, cell division, chromosome partitioning
Y	0	0.0	Nuclear structure
V	55	1.9	Defense mechanisms
T	146	5.1	Signal transduction mechanisms
M	278	9.7	Cell wall/membrane/envelope biogenesis
N	11	0.4	Cell motility
Z	0	0.0	Cytoskeleton
W	0	0.0	Extracellular structures
U	47	1.6	Intracellular trafficking, secretion, and vesicular transport
O	106	3.7	Posttranslational modification, protein turnover, chaperones
C	157	5.5	Energy production and conversion
G	282	9.8	Carbohydrate transport and metabolism
E	172	6.0	Amino acid transport and metabolism
F	69	2.4	Nucleotide transport and metabolism
H	128	4.5	Coenzyme transport and metabolism
I	86	3.0	Lipid transport and metabolism
P	195	6.8	Inorganic ion transport and metabolism
Q	41	1.4	Secondary metabolites biosynthesis, transport and catabolism
R	355	12.4	General function prediction only
S	238	8.3	Function unknown
-	1,277	32.6	Not in COGs

## Insights into the genome sequence

An estimate of the overall similarity between *Pedobacter heparinus* and *P. saltans* [1] was generated with the GGDC-Genome-to-Genome Distance Calculator [49,50]. This system calculates the distances by comparing the genomes to obtain high-scoring segment pairs (HSPs) and interfering distances from a set of three formulae (1, HSP length / total length; 2, identities / HSP length; 3, identities / total length). The comparison of *P. heparinus* and *P. saltans* revealed that an average of only 4.7% of the two genomes are covered with HSPs. The identity within these HSPs was 82.3%, whereas the identity over the whole genome was 3.8%.

**Figure 4:**
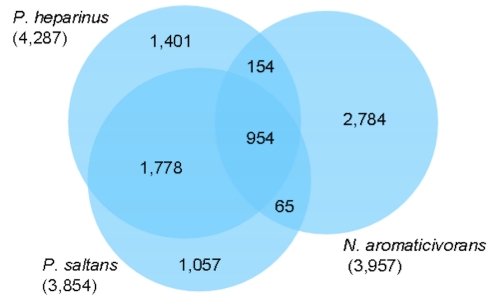
Venn diagram depicting the intersections of protein sets (total number of derived protein sequences in parentheses) of *P. heparinus*, *P. saltans* and *N. aromaticivorans*.

The fraction of shared genes in the genomes of *P. heparinus*, *P. saltans* and *Novosphingobium aromaticivorans* [51] is shown in a Venn diagram (Figure 4). The phyogentically distant reference genome of  *N.* *aromaticivorans* was selected based on its similar genome size and due to a lack of complete reference type strain genomes from the *Sphingobacteriaceae.* The numbers of pairwise shared genes were calculated with the phylogenetic profiler function of the IMG ER platform [48]. The homologous genes within the genomes were detected with a maximum E-value of 10^-5^ and a minimum identity of 30%. Only about one quarter of all genes (954 genes) are shared by all three genomes, whereas the two *Pedobacter* species share 2,732 genes, corresponding to 63.7% (*P. heparinus*) and 70.9% (*P. saltans*) of their genes. The pairwise comparison of *N. aromaticivorans* with the two *Pedobacter* species revealed only 154 (*P. heparinus*) and 65 (*N. aromaticivorans*) homologous genes (Figure 4).

Among those genes that are shared by the three genomes, are those which might be responsible for the yellow color of the organisms. These genes encode enzymes that are involved in the synthesis of carotenoids. Biosynthesis of carotenoids starts with geranylgeranyl pyrophosphate synthases combining farnesyl pyrophosphate with C_5_ isoprenoid units to C_20_-molecules, geranylgeranyl pyrophosphate. The phytoene synthase catalyzes the condensation of two geranylgeranyl pyrophosphate molecules followed by the removal of diphosphate and a proton shift leading to the formation of phytoene. Sequential desaturation steps are catalyzed by phytoene desaturase followed by cyclization of the ends of the molecules catalyzed by the lycopene cyclase [52]. Genes encoding lycopene cyclases (Phep_2088, Pedsa_2222, Saro_1817) and phytoene synthases (Phep_2092, Pedsa_2218, Saro_1814) were identified in the genomes. In the two *Pedobacter* species, genes coding for phytoene desaturases (Phep_2093, Pedsa_2217) were also identified. A carotene hydroxylase gene (Saro_1168) was only identified in the genome of *N. aromaticivorans*.

As the two *Pedobacter* species are known for their ability to degrade heparin, it is not surprising that the genomes encode several heparinase encoding genes: seven (*P. saltans*) and five (*P. heparinus*) heparinases, were identified, whereas *N. aromaticivorans* encodes only one heparinase.

Fucoidan degradation was not determined experimentally, but is assumed as both *P. saltans* and *P. heparinus* have genes for eleven and ten α-fucosidases respectively. In addition, 12 (*P. saltans*) and 18 (*P. heparinus*) α-sulfatases genes were identified, whereas *N. aromaticivorans* contains only five α-sulfatases and no α-fucosidase genes. Experimental evidence for the fucoidan hydrolysis in *Pedobacter* has not been found, but for *Mucilaginibacter paludis* and *M. gracilis,* which are also members of the family *Sphingobacteriaceae,* have been experimentally confirmed to exhibit fucoidan degradation [53]. Moreover, Sakai *et al*. [54] reported the existence of intracellular α-L-fucosidases and sulfatases, which enable ‘*F. fucoidanolyticus’* to degrade fucoidan.
